# Postoperative Delirium is Associated with Negative Outcomes and Long-Term Mortality in Elderly Koreans: A Retrospective Observational Study

**DOI:** 10.3390/medicina55100618

**Published:** 2019-09-20

**Authors:** Eun A Park, Min Young Kim

**Affiliations:** 1 Department of Nursing, Pukyong National University, Busan 48513, Korea; soundness@pknu.ac.kr; 2 Department of Nursing, Ulsan University, Ulsan 44610, Korea

**Keywords:** delirium, surgery, outcome, mortality, elderly

## Abstract

*Background and objectives:* Delirium is an acute state that causes confusion and occurs frequently after surgery in elderly patients. Delirium is also related to various clinical complications. With increasing numbers of surgeries performed on elderly Koreans, the number of cases of delirium and associated complications will likely rise. The purpose of the present study was to determine whether postoperative delirium in elderly Korean patients negatively influenced other clinical outcomes and their long-term mortality. *Materials and Methods:* The medical records of 1016 elderly patients (65 years or older) who underwent major abdominal surgery from January 2014 to December 2016 were retrospectively investigated. To determine long-term mortality, patients were followed for up to 12 months post-operation. *Results:* Delirium occurred in 194 patients (18.3%). Postoperative delirium was significantly associated with the length of hospital stay (B = 2.72), length of ICU stay (B = 18.78), adverse medical events (OR = 2.26, CI = 1.45–3.52), reoperation (OR = 5.50, CI = 1.66–18.22), ICU readmission (OR = 14.10, CI = 2.97–66.90), medical costs (B = 2473.85), discharge to somewhere other than the patient’s home (OR = 6.01, CI = 3.35–10.76), hospital readmission (OR = 2.73, CI = 1.45–5.14), in-hospital mortality (OR = 3.34, CI = 1.21–9.19), three-month mortality (HR = 3.22, CI = 1.27–8.14), six-month mortality (HR = 2.85, CI = 1.28–6.36), and 12-month mortality (HR = 2.19, CI = 1.10–4.32). *Conclusions:* Postoperative delirium in elderly Korean patients was associated with negative clinical outcomes and mortality. For rapid recovery and increased survival rates in surgical patients, effective delirium-prevention care and active delirium treatments are necessary.

## 1. Introduction

Delirium involves rapid cognitive changes which occur in a short period of time and are provoked by environment changes [1]. Major predisposing factors for delirium include old age, cognitive disorders, comorbidity, and health status. Precipitating factors, such as hospitalization, surgery, and medication administration, further increase the risk of delirium [2]. Surgical patients are at a particularly elevated risk of delirium because they are exposed to multiple precipitating factors, such as anesthesia, infection, medication administration, invasive treatments, pain, and admission to an intensive care unit.

The prevalence of postoperative delirium ranges from 5.0% to 50.0%. The incidence of delirium is particularly high in elderly patients. For persons over 65 years of age, prevalence is above 30.0%, whilst for persons over 80 years of age, it ranges from 33.5% to 50.0% [3,4,5,6,7,8,9,10,11,12]. More than 80.0% of delirium cases occur one-three days post-surgery and are temporary [13,14]. However, delirium symptoms persist for more than four days in approximately 10.0% of cases [9,14,15]. Patients with delirium have temporary symptoms, such as cognitive and physical dysfunction [3,15,16], and also negative outcomes, such as increased hospital stay lengths and mortality rates [6,8,9,10]. Compared to those without delirium, elderly patients with post-operative delirium exhibit reduced cognitive functioning, poorer engagement in activities of daily living after their discharge [3,15], higher rates of readmission [8], up to three times higher in-hospital mortality, and 17.0% increases in six-month mortality rates [9,13,17]. The length of hospital stays for patients with delirium is also 1.5 times longer than that of non-delirium patients [6,17], while the medical costs related to delirium in elderly patients are reported to range from $38 to $152 billion annually [18]. Despite previous studies on delirium and postoperative outcomes, research on the independent associations between delirium and surgery-related factors is insufficient for the Korean population. Thus, additional studies of these independent relationships are required.

The elderly population in Korea (65 age ≥ has increased by 64.3% from 2007 to 2017 (4,365,218 to 7,171,227), during which time the number of surgeries done on elderly patients also increased by 100% (359,911 in 2008 to 753,557 in 2017) [19]. With this increase in elderly surgical patients in Korea, the rates of delirium and associated complications are also likely to increase. The purpose of the present study was thus to identify the degree to which delirium in elderly patients in Korea affects their postoperative outcomes and long-term mortality.

## 2. Materials and Methods

### 2.1. Design

This study used a retrospective, chart review design to investigate the effects of delirium on postoperative outcomes and long-term mortality in elderly Korean patients who underwent abdominal surgery.

### 2.2. Sample and Setting

Research subjects were patients older than 65 who were hospitalized in a single medical center (with a capacity of 900 beds or more) and underwent abdominal surgery. The abdominal surgeries in this study included both laparoscopy and open surgery performed by general surgery (including colorectal surgery, gastrointestinal tract surgery, hepatobiliary pancreatic surgery, and cancer surgery). Those who were hospitalized for at least five days after undergoing two hours or more of abdominal surgery with anesthesia were selected. Records from patients who did not consent to the use of their medical information were excluded because their records could not be viewed. Patients with communication deficits due to dementia, severe cognitive impairments, neurophysiological illnesses, pre-operative delirium symptoms, difficult/untenable consciousness assessments due to two or more days of sedation following surgery, and those with missing post-surgical delirium assessment records were excluded. Patients seen during a three-year time period from January 2014 to December 2016 were included. Among the 2104 subjects initially selected for inclusion, chart review was impossible for 372 owing to difficulties in reviewing their electronic medical records. An additional 146 patients were excluded due to missing delirium records, as were another 525 subjects who met other exclusion criteria. The electronic medical records of a total of 1061 patients were analyzed.

### 2.3. Data Collection

Data were collected using the appropriate surgery code, operation dates, and discharge dates. Delirium records from the day of the operation to five days after the operation were investigated. Outcomes for up to 12 months post-operation were investigated. Subjects’ general characteristics, illnesses and medication characteristics, operation records, and post-operative outcomes were collected from their admission notes, operation/anesthesia records, progress notes, discharge summaries, and outpatient records. Data on whether delirium occurred was collected from nursing, progress, consultation, and psychiatric consult notes.

### 2.4. Ethical Considerations

Study procedures were reviewed and approved of by the institutional ethics committee of Dong San Medical Center Hospital (IRB No. 2015-06-043 approved on 2015.06.30). Search queries during data collection included operation codes and patient age. Personal information was not collected. All collected data were stored in an encrypted electronic file. All study protocols were performed in accordance with the Helsinki Declaration.

### 2.5. Instruments

#### 2.5.1. Delirium Diagnosis

Delirium was assessed via the confusion assessment method (CAM) [20] three times daily from the day of the operation to five days post-operation, as well as every time a change in the patient’s state of consciousness was observed by a nurse and physician. If CAM results were positive or delirium was suspected, a delirium diagnosis was subsequently confirmed by a psychiatrist.

#### 2.5.2. Patients Characteristics

Characteristics tested for their association with delirium included general patient characteristics, comorbidities and medications, and surgical factors. General characteristics included age, gender, physical activity, alcohol consumption, hearing impairments, and visual impairments. Comorbidity and medication factors included the number of comorbidities, number of medications, and any antipsychotic usage (including benzodiazepine). Surgical factors included use of general anesthesia, American Society of Anesthesiologists Physical Status (ASA) scores, operative time, emergency surgery, intensive care unit (ICU) admission post-surgery, and pre-operative infection.

#### 2.5.3. Postoperative Clinical Outcomes

The postoperative clinical outcomes assessed included the length of postoperative hospital stay, any adverse events during this period, and events until 12 months after discharge. Adverse events included adverse medical events, reoperation and ICU readmission, discharge to a place other than the patient’s home, and medical costs. Post-discharge readmissions and emergency room visits were also assessed. In-hospital, 30-day, three-months, six-months, and 12-month mortality were also investigated.

### 2.6. Statistical Analyses

Data analyses was performed using SPSS 20.0 (IBM SPSS Inc., Chicago, IL, USA). For patient characteristics and postoperative clinical outcomes, means and standard deviations and numbers and percentages were reported. Statistical comparisons were made with t-, Mann-Whitney U, Chi-square, or Fisher’s exact tests, as appropriate. Multivariate analyses and logistic, liner, and Cox proportional hazards regression analyses were performed, with post-operative clinical outcomes and delirium as covariates. A Kaplan-Meier survival analysis was used to assess patient survival over time. A *p* value of <0.05 was regarded as statistically significant.

## 3. Results

Baseline postoperative delirium characteristics of our study sample are in Table 1. The average age of patients with delirium was 74.6 years old and that of those without delirium was 69.0 years old, significantly higher(*p* < 0.001). Low physical activity (49.0% and 6.6%, *p* < 0.001), alcoholism (21.1% and 10.1%, *p* < 0.001), and hearing impairments (18.6% and 3.1%, *p* < 0.001) were significantly higher in patients with delirium compared to those without, respectively. The number of comorbidities (2.6 and 1.5, *p* < 0.001), medications (6.9 and 3.6, *p* < 0.0001), and antipsychotics (40.7% and 15.9%, *p* < 0.0001) was also significantly higher among patients with delirium. An ASA score of 3 or above (40.2% and 21.6%, *p* < 0.001), emergency surgery (51.5% and 13.7%, *p* < 0.001), ICU admission after surgery (93.8% and 57.2%, *p* < 0.001), and preoperative infection (22.7% and 2.7%, *p* < 0.001) were also significantly more common in patients with delirium. Gender, visual impairments, general anesthesia use, and operative time did not differ significantly.

The postoperative clinical outcomes of both groups of patients are shown in Table 2. The length of hospital stay after surgery was significantly longer for patients with delirium (19.1 days) than those without (14.2 days) (*p* < 0.001). The length of ICU stay in patients who were admitted to the ICU after surgery was longer in those with delirium (54.4 hours) than those delirium (27.5 hours) (*p* < 0.001). Postoperative adverse event rates also differed between the groups. Medical adverse events were more frequent in patients with delirium (31.4%) than in patients without (9.5%) (*p* < 0.001), as was the case for reoperation (3.1% and 0.6%, *p* = 0.007). ICU readmissions were more common in patients with delirium (8.2%) than in those without (0.2%) (*p* < 0.001). The proportion of patients discharged to somewhere other than home was significantly higher among those with delirium (23.2%) than those without (2.7%) (*p* < 0.001). Hospital Costs were significantly higher for patients with delirium (12816.6 KRW on average) than for those without (9292.8 KRW on average) (*p* < 0.001). Readmission after discharge was significantly more common for patients with delirium (10.9%) than for those without (5.2%) (*p* = 0.004). There were no differences in emergency room visit rates between the groups.

Critically, in-hospital mortality was significantly higher among patients with delirium (7.2%) than among those without (0.9%) (*p* < 0.001). Post-operative 30-day mortality was also significantly higher in patients with delirium (5.7%) than in patients without (1.8%) (*p* = 0.005), as was three-month mortality (8.2% and 2.4%, *p* < 0.001), six-month mortality (10.3% and 2.1%, *p* < 0.001), and 12-month mortality (10.8% and 3.7%, *p* < 0.001).

Multivariate analyses revealed effects of delirium on postoperative outcomes, as shown in Table 3. Patients with delirium had significantly different hospital stay lengths (B = 2.72, *p* = 0.001), ICU stay lengths (B = 18.78, *p* < 0.001), adverse medical event rates (OR = 2.26, CI = 1.45–3.52, *p* < 0.001), reoperation rates (OR = 5.50, CI = 1.66–18.22, *p* = 0.005), ICU readmission rates (OR = 14.10, CI = 2.97–66.90, *p* = 0.001), rates of discharge to a place other than home (OR = 6.01, CI = 3.35–10.76, *p* < 0.001), medical costs (B = 2473.85, *p* < 0.001), and readmission rates (OR = 2.73, CI = 1.45–5.14, *p* = 0.002). In-hospital mortality (OR = 3.34, CI = 1.21–9.19, *p* = 0.020), three-month mortality (HR = 3.22, CI = 1.27–8.14, *p* = 0.014), six-month mortality (HR = 2.85, CI = 1.28–6.36, *p* = 0.011), and 12-month mortality (HR = 2.19, CI = 1.10–4.32, *p* = 0.025) were also significantly different. However, 30-day mortality was not significantly different. Logistic, linear, and Cox proportional hazards regressions were used. For all analyses, age, low physical activity, number of comorbidities, number of medications, general anesthesia use, ASA score ≥3, operative time, emergency surgery, ICU admission after surgery, and preoperative infection were applied as covariates.

**Figure 1:**
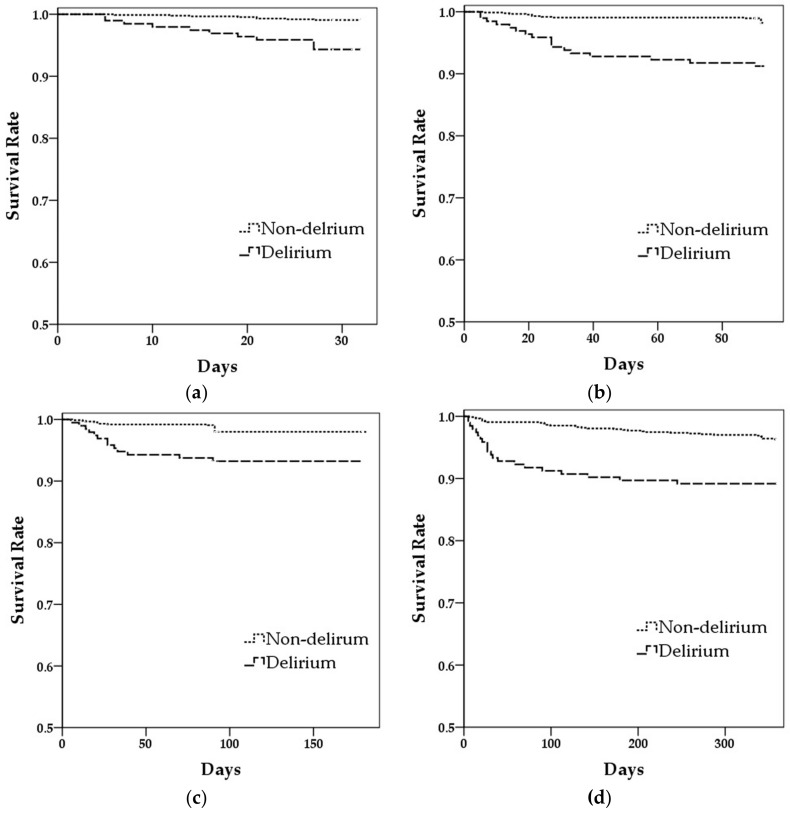
Kaplan-Meier survival curves for mortality with postoperative delirium. (**a**) thirty-day mortality; (**b**) three- month mortality; (**c**) six-month mortality; (**d**) twelve-month mortality.

Survival rate analyses are as shown in Figure 1. The 30-day (log-rank = 20.62, *p* < 0.001), three-month (log-rank = 37.25, *p* < 0.001), six-month (log-rank = 32.09, *p* < 0.001), and 12-month (log-rank = 18.15, *p* < 0.001) survival of patients with delirium was significantly lower than for those without.

## 4. Discussion

The present study assessed the effect of postoperative delirium in the elderly on their postoperative outcomes and long-term mortality. The length of hospital stays among patients with delirium was four days longer on average, and ICU stay lengths 24 hours longer, than for patients without delirium. Other studies on postoperative delirium have shown that the average length of stay for spine surgery patients was three days or longer [6,8,10], the average for hip surgery patients was two days or longer [9], the average for cardiac surgery patients was four days or longer [12], the average for acute care surgery patients was seven days or longer [4], and the average for surgical trauma ICU and major surgery patients was four days or longer [21,22]. The length of hospital stays tends to increase with post-operative delirium status.

Longer hospital stays can lead to adverse complications, such as increased in-hospital infection risk, limited activity, and a delayed return to activities of daily living, as well as an increased caregiver burden on families. Indeed, the present study revealed that delirium affected rates of patient discharge to a place other than their home, as well as readmission after discharge. The results of a 24-month follow-up with elderly patients who underwent hip surgery revealed that the activities of daily living among patients with delirium were reduced by more than 1.7-fold [3], while their cognitive functioning after major surgery decreased by more than four-fold [15]. Furthermore, readmission rates and rates of discharge to places other than home among patients with delirium after spine surgery increased by more than three-fold [8] and 1.8-fold [6,23], respectively. These results, together with our own, strongly suggest that delirium affects patients’ return to activities of daily living. Most surgical patients wish to recover their health promptly after surgery and return to their daily lives as quickly as possible. However, readmission to another institution and long-term hospital stays can delay patient normalization post-operation and expose them to additional risks. Postoperative delirium in the elderly is therefore a significant cause of long-term health problems.

The present study revealed that postoperative delirium also increased medical costs. Another study of patients with delirium who underwent major urologic surgery reported that medical costs increased by 2697$ per person [24]. Another study that evaluated the medical records of patients who underwent liver transplantation surgery also found that preventing delirium is economically efficient, given that the cost of delirium treatment was much more than the cost of its delirium prevention [25]. In Korea, the proportion of medical insurance paid for by the government is high. Because delirium not only increases individual healthcare costs but also national ones, the Ministry of Health and Welfare and medical professionals should actively engage in its prevention.

Delirium also affects postoperative adverse events, as found here and elsewhere. The prevalence of adverse medical events, such as infections and respiratory problems, as well as reoperation and ICU readmissions, were higher among patients with delirium in the present study. Internal medicine complications, such decreased systolic blood pressure, dehydration, malnutrition, pressure sores, and respiratory problems, as well as external medicine complications, such as wound infections and reoperation, are increased in patients with delirium [8,10,12,22]. Delirium affects these outcomes because hyperactive delirium often involves patients self-removing medical devices, hyperventilation, and violent behaviors, which can destabilize the patient’s condition. This destabilization can disrupt vital signs, and sedation used to calm patients can cause additional problems. Internal and external medicine complications that occur after surgery not only result in delayed recovery, but also affect mortality rates. Unexpected and negative outcomes in surgical patients may also occur. Therefore, active treatment and prevention are important for the prevention of postoperative delirium and/or its effects on patient outcomes.

The present study found that the postoperative delirium affects both short-term and long-term mortality. The in-hospital mortality of patients with delirium was more than three times higher than for those without delirium, an increase that is similar to previous studies of orthopedic and major surgery patients [13,24]. These results also demonstrate that delirium is an important predictor of patient outcomes because it can cause not only delayed postoperative recovery, but also increased in-hospital mortality rates. Furthermore, delirium had a more than two-fold effect on three-, six-, and 12-month postoperative mortality rates in the present study. This echoes previous studies which have also found that the six-month mortality after elective and hip surgeries was more than two times [23,26] but less than six times higher [22] in patients with delirium, though covariates were not considered. Thus, explaining the independent relationship between delirium and mortality is somewhat difficult.

Another study of 12-month and 10-year mortality after hip surgery found no significant correlation with delirium [27,28]. This is likely because subjects in the present study had dementia and cognitive impairments, factors which affect delirium’s diagnosis and mortality. However, a review of postoperative delirium effects on mortality found that delirium up to an average of 20 months post-operation had a significant effect on survival [26]. Together, these results and our own suggest that postoperative delirium is associated with patient mortality, even after discharge.

Delirium is an acute cognitive dysfunction, defined as a mental disorder with various symptoms and a short duration [1]. Although the duration of delirium onset is typically short (1–3 days), the negative impacts of delirium are diverse and may appear over a long period of time. Interestingly, Korean surgical professionals have recently taken an interest in delirium. However, these professionals often prioritize surgical complications and outcomes over secondary symptoms, such as delirium. For rapid recovery of elderly surgical patients and assisting with their return to healthy daily life, careful observation, prevention, and active intervention for postoperative delirium are necessary.

The present study screened for delirium for five days post-surgery and consulted a psychiatrist for delirium diagnoses. Thus, it has the methodological strength of using relatively accurate delirium diagnosis data. Furthermore, we assessed the long-term survival of surgical patients by investigating their records for up to 12 months post-surgery. Despite this study’s strengths, however, it has a number of limitations which warrant some discussion. First, CAM delirium diagnostic instruments are limited because they are insufficient to diagnose hypoactive delirium. Thus, it is necessary to use a supplementary delirium diagnostic instrument. Second, there is a limitation in the interpretation of the results due to the lack of classification of the surgical methods and the various abdominal surgeries, and further research is necessary which accounts for surgical technique and type of surgery. Finally, the current study used patients who underwent abdominal surgery in a single medical center. Therefore, caution is needed before our results can be generalized more broadly. In the future, multicenter studies of patients who underwent a variety of different surgeries are necessary.

## 5. Conclusions

The present study investigated the effect of postoperative delirium in the elderly on postoperative outcomes and long-term mortality. In elderly patients with postoperative delirium, hospital and ICU stays were longer, medical costs were higher, and patients experienced other adverse events. Patients with delirium also had higher rates of readmission and of discharge to places other than their homes. Critically, the mortality among patients with delirium was also increased for up to 12 months post-operation. These results indicate the importance of effective delirium prevention and active delirium treatment strategies for rapid recovery and improved survival rates in elderly post-operative patients.

## Figures and Tables

**Table 1:** Patient characteristics (*n* = 1061).

Variable	Delirium (*n* = 194)	Non-Delirium (*n* = 867)	*p*
	*n* (%) or Mean (Range)	*n* (%) or Mean (Range)	
**General characteristics**			
Age (years)	74.6 (60–91)	69.0 (60–95)	<0.001
Male	126 (64.9)	567 (65.4)	0.905
Low physical activity ^1^	95 (49.0)	57 (6.6)	<0.001
Alcoholism	41 (21.1)	88 (10.1)	<0.001
Hearing impairment	36 (18.6)	27 (3.1)	<0.001
Visual impairment	34 (17.5)	164 (18.9)	0.653
**Co-morbidity and medication**			
Number of comorbidities	2.6 (0–7)	1.5 (0–7)	<0.001
Number of medications	6.9 (0–19)	3.6 (0–16)	<0.001
Antipsychotics	79 (40.7)	138 (15.9)	<0.001
**Surgical factors**			
General anesthesia use	181 (93.3)	836 (96.4)	0.070
ASA score ≥3	78 (40.2)	187 (21.6)	<0.001
Operative time (min)	239.2 (60–770)	240.6 (40–750)	0.052
Emergency surgery	100 (51.5)	119 (13.7)	<0.001
ICU admission after surgery	182 (93.8)	496 (57.2)	<0.001
Preoperative Infection	44 (22.7)	23 (2.7)	<0.001

ASA, The American society of anesthesiologists physical Status; ICU, intensive care unit; *p* < 0.05; ^1^ Need assistance for daily living.

**Table 2:** Postoperative clinical outcomes and mortality (*N* = 1061).

Variable	Delirium (*n* = 194)	Non-Delirium (*n* = 867)	*p*
	*n* (%) or Mean (Range)	*n* (%) or Mean (Range)	
**Length of stay**			
Hospital stay length after surgery (days)	19.1 (5–60)	14.2 (4–94)	<0.001
ICU stay length (hours)	54.4 (7–714)	27.5 (8–460)	<0.001
**Adverse Events**			
Adverse medical events	61 (31.4)	82 (9.5)	<0.001
Reoperation	6 (3.1)	5 (0.6)	0.007
ICU readmission	16 (8.2)	2 (0.2)	<0.001
**Discharge**			
Discharge to a place other than home	45 (23.2)	23 (2.7)	<0.001
Costs (Korean dollars × 10^3^)	12816.3 (755–73168)	9292.8 (498–75270)	<0.001
**After discharge**			
Readmission	19 (10.9)	44 (5.2)	0.004
ER visit	5 (2.9)	26 (3.1)	0.899
**Mortality**			
In-hospital mortality	14 (7.2)	8 (0.9)	<0.001
30-day mortality	11 (5.7)	16 (1.8)	0.005
Three-month mortality	16 (8.2)	21 (2.4)	<0.001
Six-month mortality	20 (10.3)	18 (2.1)	<0.001
12-month mortality	21 (10.8)	32 (3.7)	<0.001

ER, emergency rooms; *p* < 0.05.

**Table 3:** Clinical outcomes and mortality among postoperative delirium patients, identified by multivariate analyses ^1^ (*n* = 1061).

Variable	B	OR/HR (CI)	SE	β	t	*p*
**Length of stay**						
Hospital stay length (days) ^2^	2.72		0.81	0.11	3.36	0.001
ICU stay length (hours) ^2^	18.78		3.77	0.19	4.97	<0.001
**Adverse events**						
Adverse medical events ^3^	0.81	2.26 (1.45–3.52)				<0.001
Reoperation ^3^	1.71	5.50 (1.66–18.22)				0.005
ICU readmission ^3^	2.65	14.10 (2.97–66.90)				0.001
**Discharge**						
Discharge to a place other than home ^3^	1.79	6.01 (3.35–10.76)				<0.001
Costs (Korean dollars × 10^3^) ^2^	2473.85		513.22	0.14	4.82	<0.001
**After discharge**						
Readmission ^3^	1.00	2.73 (1.45–5.14)				0.002
ER visit ^3^	−0.88	0.42 (0.14–1.23)				0.114
**Mortality**						
In-hospital mortality ^3^	1.21	3.34 (1.21–9.19)				0.020
30-day mortality ^4^	0.99	2.68 (0.96–7.46)				0.157
Three-month mortality ^4^	1.17	3.22 (1.27–8.14)				0.014
Six-month mortality ^4^	1.05	2.85 (1.28–6.36)				0.011
12-month mortality ^4^	0.79	2.19 (1.10–4.32)				0.025

B, the unstandardized beta; β, the standardized beta; CI, confidence interval; ER, emergency rooms; HR, hazard ration; ICU, intensive care unit; OR, odds ratio; SE, standard error; *p* < 0.05. ^1^ Adjusted covariate variables: age, low physical activity, number of comorbidities, number of medications, general anesthesia use, ASA score ≥3, operative time, emergency surgery, ICU admission after surgery, preoperative infection.^2^ Linear regression; ^3^ Logistic regression; ^4^ Cox proportional hazards regression.
